# Fabrication of Porous Lead Bromide Films by Introducing Indium Tribromide for Efficient Inorganic CsPbBr_3_ Perovskite Solar Cells

**DOI:** 10.3390/nano11051253

**Published:** 2021-05-11

**Authors:** Xianwei Meng, Kailin Chi, Qian Li, Bingtao Feng, Haodi Wang, Tianjiao Gao, Pengyu Zhou, Haibin Yang, Wuyou Fu

**Affiliations:** 1 State Key Laboratory of Superhard Materials, College of Physics, Jilin University, Changchun 130012, China; xwmeng17@mails.jlu.edu.cn (X.M.); fengbt20@mails.jlu.edu.cn (B.F.); yanghb@jlu.edu.cn (H.Y.); 2 School of Science, Northeast Electric Power University, Jilin 132012, China; 20162715@neepu.edu.cn; 3 Beijing Key Lab of Cryo-Biomedical Engineering and Key Lab of Cryogenics, Technical Institute of Physics and Chemistry, Chinese Academy of Sciences, Beijing 100190, China; liqian@mail.ipc.ac.cn; 4 College of Physics, Jilin University, Changchun 130012, China; hdwang20@mails.jlu.edu.cn (H.W.); gaotj19@mails.jlu.edu.cn (T.G.)

**Keywords:** CsPbBr_3_, perovskite solar cells, InBr_3_, PbBr_2_, incorporation

## Abstract

In the process of preparing CsPbBr_3_ films by two-step or multi-step methods, due to the low solubility of CsBr in organic solvents, the prepared perovskite films often have a large number of holes, which is definitely not conducive to the performance of CsPbBr_3_ perovskite solar cells (PSCs). In response to this problem, this article proposed a method of introducing InBr_3_ into the PbBr_2_ precursor to prepare a porous PbBr_2_ film to increase the reaction efficiency between CsBr and PbBr_2_ and achieve the purpose of In (Ⅲ) incorporation, which not only optimized the morphology of the produced CsPbBr_3_ film but also enhanced the charge extraction and transport capabilities, which was ascribed to the reduction of the trap state density and impurity phases in the perovskite films, improving the performance of CsPbBr_3_ PSCs. At the optimal InBr_3_ concentration of 0.21 M, the InBr_3_:CsPbBr_3_ perovskite solar cell exhibited a power conversion efficiency of 6.48%, which was significantly higher than that of the pristine device.

## 1. Introduction

After more than ten years of rapid development, lead halide-based perovskite solar cells have made remarkable achievements, but they seem to be in a vicious circle where high efficiency and high stability are contradictory to each other. Although the power conversion efficiency (PCE) of organic-inorganic hybrid perovskite solar cells (PSCs) has increased from the initial 3.8% to more than 25% at present [1,2,3,4,5], yet due to the strong volatility of common A-site organic cations, such as organic methylammonium (MA^+^) and formamidinium (FA^+^), the organic components disappear under thermal stress [6,7]. In terms of thermal stability, the all-inorganic cesium-lead halide perovskite CsPbX_3_ (X: iodine or bromine), which is formed by using more stable inorganic cesium ions (Cs^+^) to completely replace organic cations, usually performs better stability [8,9,10,11] and is not prone to degradation at temperatures above 400 °C [8,12]. This provides the necessary conditions for the long-term stable use of CsPbX_3_ PSCs [13,14,15,16]. The key factor, which influences the stability of CsPbX_3_, is the moisture in the air. The presence of humidity changes the phase of the perovskite and reduces the stability of the photovoltaic device, but this does not directly cause the decomposition of CsPbX_3_ (mainly I-rich CsPbX_3_) and the lack of components [8,17,18]. Of course, this phase change is reversible when heated [19].

Compared with other Cs-based inorganic perovskites, the most prominent advantage of CsPbBr_3_ is that it has a highly stable crystal structure. Whether it is the orthorhombic γ-phase at room temperature or tetragonal β-phase and cubic α-phase when heated, the geometric structures of CsPbBr_3_ have not changed much, so the electronic structures of different phases are also relatively similar [20]. For this reason, CsPbBr_3_ is also regarded as a perovskite material that presents better stability to humidity, heat, and light at ambient temperature [2,8,21,22]. Since Kulbak et al. first prepared the CsPbBr_3_ PSCs by a two-step solution-processing method in 2015 [22], in less than ten years, the PCE of the CsPbBr_3_ based PSCs have reached more than 10% with an ultrahigh open-circuit voltage (V_OC_) of 1.62 V [23], but it still has a large distance compared with CsPbBr_3_ PSCs theoretical limit PCE of 16.4% [24] and the maximized PCE of 19.0% for inorganic CsPbI_3_ PSCs [10].

In the process of preparing CsPbBr_3_ PSCs by the solution-processing method, the solubility of CsBr in commonly used polar solvents is poor, and the concentration differences between CsBr and PbBr_2_ solutions are large, which leads to the derivative phases PbBr_2_-rich CsPb_2_Br_5_ and CsBr-rich Cs_4_PbBr_6_ in the process of the generation of CsPbBr_3_ [25]. At the same time, the thickness of the non-optimized prepared perovskite film is low, and the ability to absorb light is inadequate; numerous pinholes appear in the film [26]. Consequently, a decrease appears in the PCE of CsPbBr_3_ PSCs. Regardless of whether it is a two-step sequential deposition or a multi-step method to prepare CsPbBr_3_, it is necessary to deposit PbBr_2_ first and then use CsBr to convert PbBr_2_ to CsPbBr_3_. Improving the PbBr_2_ film preparation process and adjusting the PbBr_2_ preparation method can achieve the goals of enhancing the reaction efficiency of the precursor, accurately controlling the subsequent growth of CsPbBr_3_ crystals, and reducing the generation of by-products, and finally obtain perovskite film with a high purity phase, large grain size, and high coverage [23,27]. By precisely regulating the film-forming temperature and pore diameter of the PbBr_2_ precursor film, Zhao et al. [23] minimized the compressive stress of the perovskite film and prepared CsPbBr_3_ grains with a size of up to 1.62 μm, which not only made the PCE of the all-inorganic CsPbBr_3_ perovskite solar cell reach 10.7%, the open-circuit voltage (V_OC_) as high as 1.6 V, and it also kept the device extremely stable in a high-humidity air environment. Lee et al. [28] introduced CZISSE QDs quantum dots into the PbBr_2_ film. CZISSE QDs acted as seeds to promote the crystallization of CsPbBr_3_ and, at the same time, penetrated into the m-TiO_2_ and CsPbBr_3_ perovskite films to increase the electron extraction and transportability of TiO_2_, thereby improving the conversion efficiency of the device by 20.6%.

In this work, InBr_3_ was introduced into the PbBr_2_ precursor solution, so that the multiple ordered crystal orientations of lead bromide grew, and the original rough and extremely uneven grain distribution of the PbBr_2_ film evolved into a large uniform-porous film with pores. This morphological change ensured the full diffusion and uniform reaction of CsBr in the PbBr_2_ film during the synthesis of CsPbBr_3_ and was conducive to the formation of polycrystalline surface growth, high purity phase, and uniform morphology InBr_3_: CsPbBr_3_ film. The PCE of the small area (0.09 cm^2^) InBr_3_:CsPbBr_3_ PSC obtained after conditions optimization was 6.48%, in particular, the V_OC_ was significantly improved.

## 2. Experiment Section

### 2.1. Materials

PbBr_2_ (99.99%) and CsBr (99.9%) were purchased from Xi’an Polymer Light Technology Corp. (Xi’an, China) and were not purified. InBr_3_ (99.9%) was purchased from Shanghai Macklin Biochemical Co., Ltd. (Shanghai, China). Titanium diisopropoxide bis (acetylacetonate; 75 wt% in 2-propanol) was purchased from Sigma-Aldrich (Louis, MO, USA). Titanium dioxide (TiO_2_) paste (18 NR-T) was purchased from Greatcell Solar Limited (Queanbeyan, Australia). N,N-Dimethylformamide (DMF, chromatographic grade, ≥99.9%), methanol (chromatographic grade, ≥99.9%), ethanol (chromatographic grade, ≥99.8%), and isopropanol (≥99.5%) were purchased from Aladdin (Shanghai, China). The fluorine-doped tin oxide coated glass (FTO, 6 Ω/□) and carbon paste were purchased from Opvtech New Energy Co., Ltd. (Yingkou, China) and Shanghai MaterWin New Materials Co., Ltd. (Shanghai, China), respectively.

### 2.2. Device Fabrication

All the following processes were carried out in a fume hood environment, without artificial control of the temperature, humidity, and airflow rate of the surrounding environment. The fluorine-doped tin-oxide-coated glasses were patterned by laser etching and cleaned by ultrasonic with acetone, isopropanol, ethanol, and deionized water. After being dried by high purity nitrogen, the FTO were further cleaned by an ultrasound treatment for 15 min and washed with ethanol. Afterward, the pre-conditioned FTO were spin-coated with 0.15 M titanium diisopropoxide bis(acetylacetonate) in 1-butanol at 5000 rpm for 20 s and were heated at 125 °C for 5 min. After these substrates returned to room temperature, the above procedure was repeated twice with 0.3 M titanium diisopropoxide bis(acetylacetonate) in 1-butanol and compact TiO_2_ (c-TiO_2_) was obtained. After that, the mesoporous TiO_2_ (m-TiO_2_) films were deposited on the above cooling c-TiO_2_ by spin-coating at 5000 rpm for 30 s by means of TiO_2_ paste diluted with ethanol. Further, the obtained layers were dried at 125 °C for 5 min followed by the muffle furnace at 500 °C for 30 min. After the muffle furnace was lowered to room temperature, pre-coated substrates were acquired.

Perovskite films were synthesized by a multistep solution-processing method. 0.03, 0.09, 0.15, 0.21, and 0.27 mmol InBr_3_ were added into 1 mL DMF of PbBr_2_ (1 M) and stirred. After the InBr_3_ was completely dissolved, the DMF mixed solution was spin-coated on pre-coated substrates at 2000 rpm for 30 s and then heated to 90 °C for 30 min. Afterward, the methanol solution of CsBr (0.07 M) was spin-coated on the InBr_3_:PbBr_2_ film at 5000 rpm for 30 s and heated to 250 °C for 5 min, and this step was repeated five times. Next, the prepared sample was placed in isopropanol and soaked for 30 min and annealed at 250 °C for 15 min to remove excess CsBr. Finally, the carbon paste was deposited coated on the perovskite films by using the doctor blade coating method and dried at 100 °C for 10 min. The effective area of the back electrode was 3 mm × 3 mm, which defined the active area of each device.

### 2.3. Characterization

The morphologies of the synthesized films and energy-dispersive X-ray spectroscopy (EDS) mapping images were observed by a scanning electron microscope (SEM, FEI MAGELLAN 400, FEI, Hillsboro, OR, USA). The crystal structure of the synthesized sample was determined by means of X-ray diffraction (XRD, Cu Kα radiation, λ = 1.5418 Å, Rigaku D/max2500, Tokyo, Japan). The steady-state photoluminescence (PL) spectra of perovskite films were performed using a Renishaw InVia micro-Raman spectroscopy system (Renishaw, Wotton-under-Edge, UK) with a 473 nm excitation source. Ultraviolet photoelectron (UPS) and X-ray photoelectron spectroscopy (XPS) were carried out by an X-ray photoelectron spectrometer (EscaLab Xi+, Thermofisher, Waltham, MA, USA). UV-Vis spectrometer (UV-3600, Shimadzu, Kyoto, Japan) was employed to measure the absorption spectrum in the range of 200 nm to 800 nm. The current-voltage (*J–V*) characteristics and the external quantum efficiency (EQE) of the fabricated solar devices were measured by a solar cells test system (XP3000, Sanyou, Beijing, China) and an EQE measured system (QTest Station 1000A, CROWNTECH, Inc., Macungie, PA, USA), respectively. The impedance was executed at 10^−1^~10^7^ Hz by using an impedance analyzer in a dark environment (Solartron 1260 coupled to the dielectric interface 1296, Farnborough, UK).

## 3. Results and Discussion

**Figure 1:**
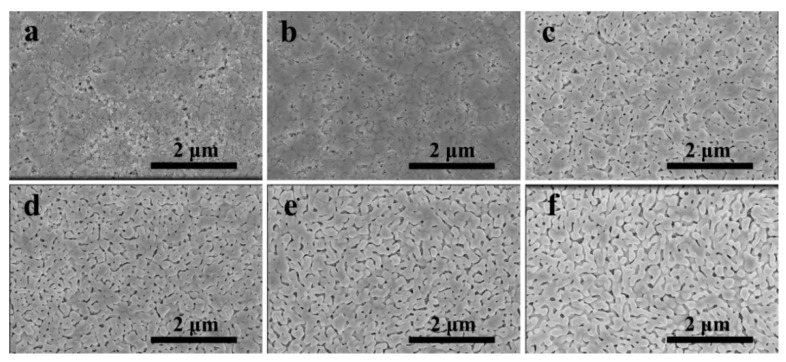
Top-view scanning electron microscope (SEM) images of PbBr_2_ films by introducing different concentrations of InBr_3_: (**a**) 0.00 M; (**b**) 0.03 M; (**c**) 0.09 M; (**d**) 0.15 M; (**e**) 0.21 M; (**f**) 0.27 M.

In the two-step or multi-step method of preparation of perovskite, the quality of the PbBr_2_ film determined the morphology of the following perovskite film. Figure 1 shows the top-view SEM images of PbBr_2_ films by introducing different concentrations of InBr_3_. When there was no InBr_3_ in the PbBr_2_ precursor solution, as shown in Figure 1a, the surface of the obtained sample was rough, and the PbBr_2_ grain distribution was extremely uneven, and a large area of exposed m-TiO_2_ could be directly observed. When the PbBr_2_ precursor solution was introduced into 0.03 M InBr_3_, as shown in Figure 1b, the surface of the PbBr_2_ film was flat, and the coverage of the m-TiO_2_ film was increased, and the observable exposed m-TiO_2_ area was significantly reduced. With the gradual increase in the concentration of introduced InBr_3_ (Figure 1c–f), the PbBr_2_ film appeared porous, but the number of pores decreased as the concentration of InBr_3_ increased. Meanwhile, the porosity volume increased as the concentration of InBr_3_ increased. From the cross-sectional view of PbBr_2_ shown in Figure S1 (Supplementary Materials), we could clearly see that the pure PbBr_2_ film has a flat surface and a uniform thickness of about 50~60 nm, and the m-TiO_2_ was filled with PbBr_2_. As the concentration of InBr_3_ introduced gradually increased, the thickness of the PbBr_2_ film also gradually increased (about 70 nm, 90 nm, 100 nm, 120 nm, 160 nm), and the film roughness increased. The above data could clearly demonstrate that the introduction of InBr_3_ could effectively affect the morphology of the PbBr_2_ film. The increase in the porosity volume, roughness, and thickness of the PbBr_2_ film facilitated the diffusion of the subsequent CsBr solution, increased the reaction efficiency with CsBr, and then achieved the full growth of CsPbBr_3_ grains [29,30].

**Figure 2:**
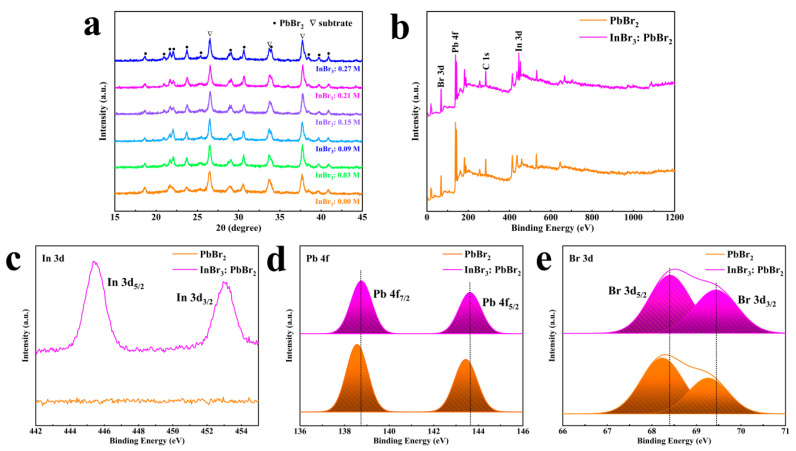
(**a**) X-ray diffraction (XRD) patterns of PbBr_2_ films by introducing different concentrations of InBr_3_. (**b**) X-ray photoelectron spectroscopy (XPS) spectra, and (**c**) In 3d, (**d**) Pb 4f, (**e**) Br 3d XPS core spectra of InBr_3_:PbBr_2_ film.

To investigate the influence of InBr_3_ on the structure of PbBr_2_, XRD patterns of InBr_3_:PbBr_2_ films are shown in Figure 2a. It could be seen that Pure PbBr_2_ was in the orthorhombic phase crystal structure (PDF#84-1181) [23]. When InBr_3_ was introduced, for all concentrations of InBr_3_ used, two new diffraction peaks of (011) and (200) crystallographic planes of PbBr_2_ could be found at 2θ of 20.94° and 22.05°, but no diffraction peak belonging to InBr_3_ or other protobromides were found. Since InBr_3_ did not exist in the form of simple In^3+^ and Br^-^ in the DMF solution, it was self-ionized and formed various complexes [31,32,33]. Therefore, we speculated that in the process of PbBr_2_ crystal growth, In (Ⅲ) could be in the form of free In^3+^ to replace a part of the Pb vacancy or exchange it with Pb, or the In cluster was directly bound to host lattice constituents [33,34,35]. Meanwhile, the PbBr_2_ crystal was made to grow along multiple ordered crystal orientations. When using the XPS technique to prove the presence of In in PbBr_2_ films, not surprisingly, characteristic peaks belonging to Br 3d, Pb 4f, and In 3d were found in the XPS spectra for the pure PbBr_2_ and InBr_3_:PbBr_2_ films, as shown in Figure 2b. According to Figure 2c–e, the core level In3d_5/2_ and 3d_3/2_ were located at 445.4 eV and 452.9 eV, respectively, and the Pb 4f_5/2_ and 4f_7/2_ peaks in Pb 4f spectrum and Br 3d_3/2_ and 3d_5/2_ peaks in Br 3d spectrum all moved towards higher binding energies, which showed that Pb-Br interactions were enhanced after In^3+^ or In cluster incorporation [36]. Additionally, the EDS mapping was also utilized to confirm the presence of In in the InBr_3_:PbBr_2_ films. Figure S2 (Supplementary Materials) demonstrated that all elements were uniformly distributed in the corresponding film, especially, there was no aggregation of In elements.

**Figure 3:**
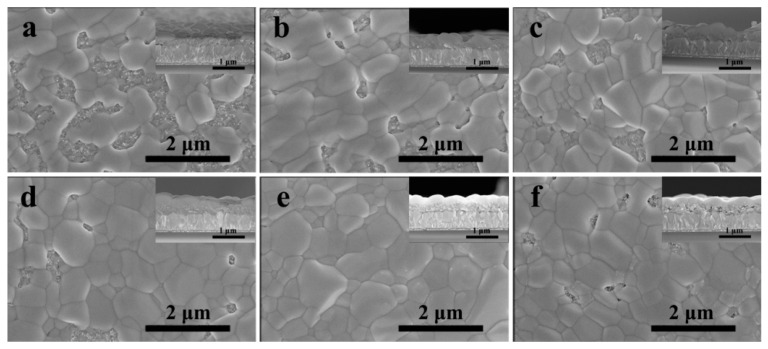
Top-view and cross-sectional (insets) SEM images of CsPbBr_3_ films by introducing different concentrations of InBr_3_: (**a**) 0.00 M; (**b**) 0.03 M; (**c**) 0.09 M; (**d**) 0.15 M; (**e**) 0.21 M; (**f**) 0.27 M.

Figure 3 depicts the top-view SEM images of perovskite films without and with InBr_3_ with the corresponding cross-section SEM images inserted in the inset. The size of the crystal grain of the pure CsPbBr_3_ was quite different, the film uniformity and coverage were also bad, and the bare m-TiO_2_ could be clearly seen. As the concentration of introduced InBr_3_ gradually increased (0.03~0.21 M), the coverage of m-TiO_2_ by CsPbBr_3_ films also gradually increased, and the size and number of pores in each film showed a downward trend. This morphological change was conducive to the performance of the perovskite cells. However, when the concentration of InBr_3_ was further increased by 0.27 M, there were again obvious holes in the CsPbBr_3_ film. This result indicated that the quality and surface CsPbBr_3_ film depended on the morphology of the corresponding porous InBr_3_:PbBr_2_ film greatly that was, the morphology of CsPbBr_3_ film could be modified by changing InBr_3_ concentration.

**Figure 4:**
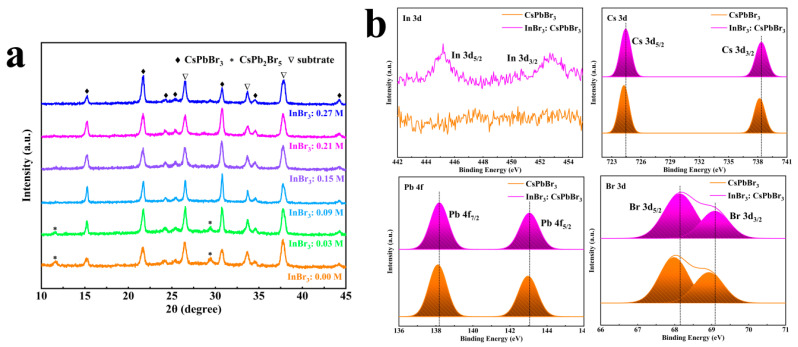
(**a**) XRD patterns of CsPbBr_3_ films by introducing different concentrations of InBr_3_. (**b**) In 3d, Cs 3d, Pb 4f, Br 3d XPS core spectra of InBr_3_:CsPbBr_3_ film.

The XRD patterns shown in Figure 4a revealed that all CsPbBr_3_ films had a cubic structure (PDF#54-0752) [23], and the positions of the diffraction peaks were not significantly shifted to high or low angles, which demonstrated that, although In cluster could promote growth along multiple ordered crystal orientations, it could not change the phase of CsPbBr_3_. When the concentration of the introduced InBr_3_ was 0.00 M and 0.03 M, there existed two peaks located at 11.7° and 29.4°, respectively, which belonged to (002) and (213) lattice planes of the CsPb_2_Br_5_ phase [37]. As the concentration of InBr_3_ was further increased (0.09~0.27 M), no obvious impurity peak belonging to CsPb_2_Br_5_ or Cs_4_PbBr_6_ phase could be observed. In fact, the control of the reaction rate between CsBr and PbBr_2_ was a necessary condition for preparing CsPbBr_3_ films with a high purity phase and high coverage. Based on Figure 1, the appropriate concentration of InBr_3_ could make the PbBr_2_ film have higher porosity, which provided more effective diffusion paths for the diffusion of CsBr methanol solution in the PbBr_2_ film, and appropriately increased the contact area between CsBr and PbBr_2_. That could also ensure the full growth of CsPbBr_3_ crystal grains and, at the same time, could prevent the formation of the impurity phase due to excessive PbBr_2_ or CsBr. However, if the concentration of the InBr_3_ introduced into PbBr_2_ was too low or too high, it was not conducive to controlling the reaction rate of CsBr and PbBr_2_. In the process of the reaction, due to the incomplete reaction of the precursors or the excessive growth of crystal grains, the morphology of the CsPbBr_3_ film was easily deteriorated, accompanied by the formation of byproducts. Further XPS was employed to certify the presence of the incorporated In^3+^ in the InBr_3_:CsPbBr_3_ film. Figure S3 (Supplementary Materials) exhibited the XPS of Cs 3d, Pb 4f, Br 3d, and In (Ш) 3d for the CsPbBr_3_ and the InBr_3_:CsPbBr_3_ films, respectively. As seen in Figure 4b, compared with CsPbBr_3_ film, two In signals corresponding to In 3d_5/2_ and 3d_3/2_ core levels were detected in InBr_3_:CsPbBr_3_ film, and Cs 3d, Pb 4f, and Br 3d all moved towards higher values, which means that the chemical state of the [PbBr_6_]^4-^ octahedral was altered and Pb-Br and Cs-Br interactions were enhanced after replacing Pb^2+^ (1.7497 Å) with In^3+^ (1.6590 Å) with a smaller ion radius accompanied by the size of the [PbBr_6_]^4-^ octahedral and the voids decreased [35]. The contraction of lattice and the enhancement of the spatial symmetry of the crystal structure caused by the incorporation of In^3+^ or In cluster could result in an efficient charge transport along with multiple directions, which perhaps was one of the important factors to improve the performance of CsPbBr_3_ cells [35,38]. The EDS mapping was used to characterize the cross-sectional of InBr_3_:CsPbBr_3_, and it was confirmed that In was evenly distributed inside the perovskite, which indicated the successful incorporation of CsPbBr_3_ by In (Figure S4, Supplementary Materials).

**Figure 5:**
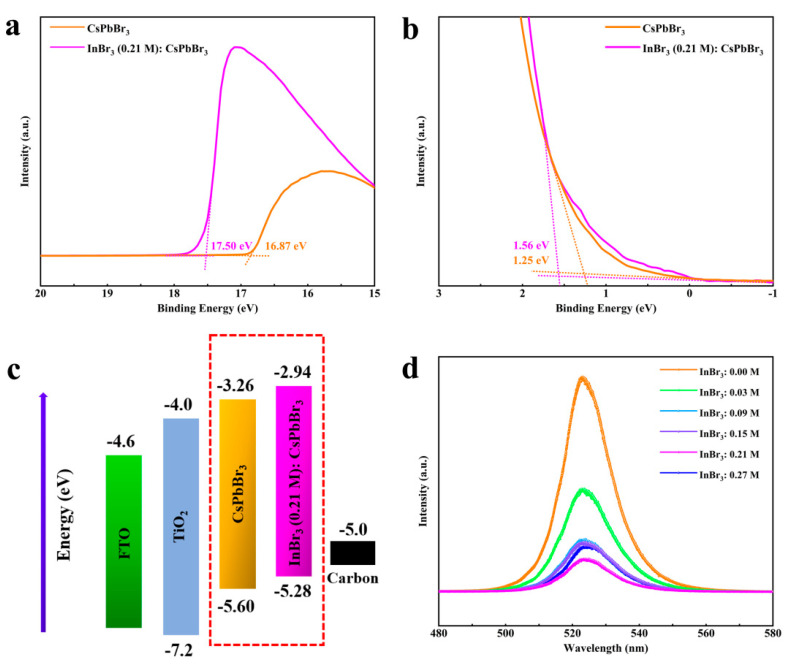
(**a**,**b**) UPS spectra of the pristine and InBr_3_ (0.21 M):CsPbBr_3_ films. The linear fittings indicate the photoemission cutoff energy boundary (E_cutoff_) and onset (E_onset_) values. (**c**) Energy level diagram for the carbon-based pristine and InBr_3_ (0.21 M):CsPbBr_3_ PSCs. (**d**) PL spectra of the cells by introducing different concentrations of InBr_3_.

Subsequently, UV-vis Spectrometer, UPS, PL were used to characterize the cells with the FTO/c-TiO_2_/m-TiO_2_/CsPbBr_3_ structure. Figure S5a (Supplementary Materials) shows the absorption spectra of the CsPbBr_3_ with different concentrations of InBr_3_. The absorption edge of each perovskite film was at approximately 530 nm within the visible region, which revealed that the concentration change of the introduced InBr_3_ did not significantly affect the light absorption range of CsPbBr_3_. Correspondingly, the calculated bandgaps (2.34 eV) did not reveal obvious and meaningful changes (Figure S5b, Supplementary Materials). As the concentration of InBr_3_ increased, so did the film’s capacity to absorb visible light. This was mainly attributed to the phase-purity of the perovskite film and the full growth of crystal grains, which was beneficial to improve the short current density (J_SC_) of the cells. The mechanism of this phenomenon was mainly attributed to the partial substitution of Pb^2+^ by In^3+^ or In cluster [34,35]. Figure 5a,b present the UPS spectra of the pristine and InBr_3_ (0.21M):CsPbBr_3_ films. By formula valence band maximum E_V__BM_ = 21.22 eV − (E_cutoff_ − E_onset_) [39,40], it could be calculated that the valence band (E_V_) of CsPbBr_3_ and InBr_3_:CsPbBr_3_ were −5.60 and −5.28 eV, respectively, which was ascribed to the rearrangement of electrons outside the Cs, Pb, and Br atoms after the incorporation of In^3+^ or In cluster [34,35]. Combined with Figure S5b, the corresponding calculated conduction band (E_C_) was −3.26 and −2.94 eV, and the energy band diagram of isolated semiconductors of the PSCs using carbon electrodes is plotted in Figure 5c [39,41]. For HTL-free PSCs, Ev of the perovskite should be deeper than the work function (W_F_) of the carbon electrode [39] so as to facilitate the extraction of photogenerated holes and reduce the energy loss of the holes during the transmission process [39]. Obviously, the incorporation of In^3+^ or In cluster effectively reduced the difference in interface energy levels, thereby facilitating the charge extraction and transfer and enhancing the photovoltaic performance of PSCs. In addition, the PL was conducted to analyze the carrier transfer behavior of CsPbBr_3_ and InBr_3_:CsPbBr_3_ films. As shown in Figure 5d, all perovskite films showed the typical emission band around 523 nm. InBr_3_ (0.21 M):CsPbBr_3_ film showed a strong quenching in contrast with the pristine and other CsPbBr_3_ films introduced with InBr_3_, which indicated that InBr_3_ could effectively inhibit the carrier recombination and enhance the charge extraction ability. The main reason behind this was that the defect density caused by the pinholes of CsPbBr_3_ films surface, and the impurity phase of CsPbBr_3_ films are obviously improved by adding InBr_3_, and the enhancement of the spatial symmetry of the crystal structure caused by partial substitution of Pb^2+^ by In^3+^ or In cluster [34,35,39,42].

**Figure 6:**
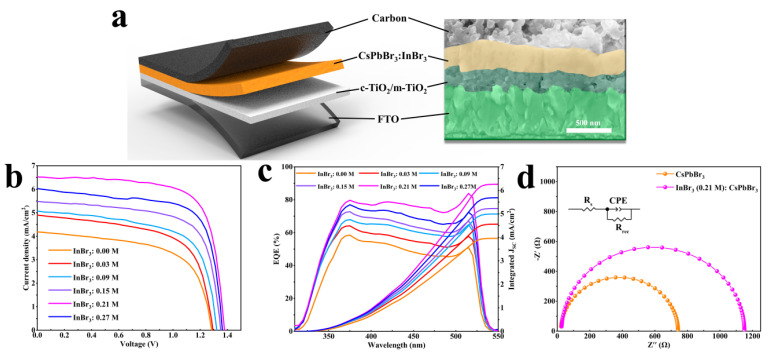
(**a**) Cross-sectional SEM image of the InBr_3_:CsPbBr_3_ device, (**b**) *J–V* characteristics, and (**c**) EQE spectra and integrated photocurrent densities for the InBr_3_:CsPbBr_3_ devices. (**d**) Nyquist plots of the pristine CsPbBr_3_ and InBr_3_ (0.21 M):CsPbBr_3_ devices with the equivalent circuit depicted in the inset.

The HTL-free PSCs were synthesized based on the standard mesoscopic architecture of c-TiO_2_/m-TiO_2_/InBr_3_:CsPbBr_3_/carbon, and the cross-section of the complete device is given in Figure 6a. The current *J–V* characteristics of relevant devices under reverse scanning are presented in Figure 6b, the corresponding forward scanning curve is shown in Figure S6 (Supplementary Materials), and the key parameters including Jsc, Voc, FF, PCE, and hysteresis index (HI) are summarized in Table 1. The PCE of all devices with InBr_3_ introduced were better than that of the pristine ones, and all parameters showed a regular trend of first increasing and then decreasing with an increase of the concentration of InBr_3_ introduced. When the concentration of InBr_3_ was 0.21 M, the corresponding device exhibited the best performance. Compared with the pristine device, the PCE of InBr_3_ (0.21 M):CsPbBr_3_ device was significantly improved from 3.29% to 6.48% with the continuously increased J_SC_ of 4.21 and 6.52 mA/cm^2^, Voc of 1.28 and 1.38 V, FF of 0.61 and 0.72, and HI of 0.25 and 0.03. When the InBr_3_ concentration was further increased to 0.27 M, the J_SC_ of the device dropped by about 0.5 mA/cm^2^, while the Voc and FF did not change significantly, which was due to the deterioration of the InBr_3_:CsPbBr_3_ film morphology. The PEC of PSCs was determined by a variety of complex factors. According to the experimental results of SEM, UV-vis, UPS, and PL, the improvements of Voc and FF were due to the rise in the energy difference between the perovskite conduction band and electron transport layer, thereby reducing the energy loss of the holes in the transmission process. The improved Jsc was not only ascribed to the quality of the InBr_3_:CsPbBr_3_ film morphology or the increase in film coverage to absorb more photons to generate more electrons but also reduced vacancy defects in the optimized CsPbBr_3_ films to improve the charge extraction and transfer process after the incorporation of In^3+^ or In cluster. In addition, the smaller hysteresis of the InBr_3_:CsPbBr_3_ cells performed than that of the pristine device might be enabled by the passivation function of InBr_3_ to diminish the defects of Pb^2+^ and Br^−^ [34,35]. Figure 6c shows the external quantum efficiency (EQE) spectrum. The highest EQE value of 84% was achieved at the InBr_3_ concentration of 0.21 M, whereas the reference devices with less or excessive InBr_3_ concentration displayed lower EQE responses. This regular change was consistent with the results of *J–V* characteristics. Additionally, the integrated current density calculated by the EQE curve of each device was very close to the J_SC_, and the mismatch was less than 5%. Figure 6d demonstrates the Nyquist plots of pristine CsPbBr_3_ and InBr_3_ (0.21 M):CsPbBr_3_ devices measured at a reverse potential of 1.0 V and the corresponding equivalent circuit model. Table S1 (Supplementary Materials) also provides a list of the fitting values of the series resistance (R_s_) and the charge recombination resistance (R_rec_). After the introduction of InBr_3_, R_rec_ increased from 765 to 1152 Ω, which showed that the incorporation of In had efficient repression of carrier recombination due to the significantly improved film formation quality of perovskites, thus reducing the trap state density and improving carriers mobility [39].

## 4. Conclusions

In the process of preparing the CsPbBr_3_ film by the multi-step method, we introduced InBr_3_ into the PbBr_2_ precursor, so that the PbBr_2_ film was transformed from a flat membrane to a porous membrane, which was beneficial to improve the reaction efficiency of CsBr and PbBr_2_, reduced the impurity in CsPbBr_3_, and optimized the surface morphology, and, finally, enabled the performance of CsPbBr_3_ PSCs to be significantly improved. When combined with host lattices, the In^3+^ or In cluster could effectively suppress the carrier recombination in the CsPbBr_3_ film and shift up the Ev of CsPbBr_3_, thereby enhancing the charge extraction and transportation capabilities. When the InBr_3_ concentration in the PbBr_2_ precursor solution was 0.21 M, the InBr_3_:CsPbBr_3_ device presented the best photovoltaic performance with a PCE of 6.48% and, especially the V_OC_ significantly increased by 100 mV compared with the pristine CsPbBr_3_. These research results confirmed that InBr_3_ has solid potentials for improving the performance of CsPbBr_3_ PSCs and also provided a reference for InBr_3_ or some other metal bromide applications in the inorganic CsPbI_3_ PSCs field and developmental direction.

## Figures and Tables

**Table 1:** Key *J–V* parameters of the InBr_3_:CsPbBr_3_.

Samples	Scan	J_SC_ (mA/cm^2^)	V_OC_ (V)	FF	PCE (%)	HI
InBr_3_: 0.00 M	Forward Reverse	4.05 4.21	1.27 1.28	0.48 0.61	2.46 3.29	0.25
InBr_3_: 0.03 M	Forward Reverse	4.82 4.87	1.27 1.29	0.53 0.62	3.24 3.90	0.17
InBr_3_: 0.09 M	Forward Reverse	5.14 5.08	1.31 1.32	0.59 0.65	3.97 4.36	0.09
InBr_3_: 0.15 M	Forward Reverse	5.45 5.49	1.33 1.35	0.64 0.68	4.63 5.04	0.08
InBr_3_: 0.21 M	Forward Reverse	6.49 6.52	1.37 1.38	0.71 0.72	6.31 6.48	0.03
InBr_3_: 0.27 M	Forward Reverse	5.95 6.01	1.35 1.37	0.66 0.70	5.30 5.76	0.08

## Supplementary Materials

The following are available online at https://www.mdpi.com/article/10.3390/nano11051253/s1, Figure S1: Cross-sectional SEM images of PbBr_2_ films by introducing different concentrations of InBr_3_: (a) 0.00 M; (b) 0.03 M; (c) 0.09 M; (d) 0.15 M; (e) 0.21 M; (f) 0.27 M; Figure S2: The SEM image of InBr_3_:PbBr_2_ film (a) and the corresponding EDS mapping of Pb (b), Br (c) and In (d); Figure S3: XPS spectra of InBr_3_:CsPbBr_3_ film; Figure S4: The cross-sectional SEM image of InBr_3_:CsPbBr_3_ film (a) and the corresponding EDS mapping of Cs (b), Pb (c), Br (d) and In (e); Figure S5: UV-vis absorption spectra (a) and (αhν)^2^ vs. hν plots (b) of the modules by introducing different concentrations of InBr_3_; Figure S6: *J*–*V* curves with forward and reverse voltage scanning for the InBr_3_:CsPbBr_3_ devices: (a) 0.00 M; (b) 0.03 M; (c) 0.09 M; (d) 0.15 M; (e) 0.21 M; (f) 0.27 M; Table S1: Electrochemical Impedance Spectroscopy parameters of the pristine and InBr_3_ (0.21 M):CsPbBr_3_ modules.
