# Supplementary material for: Fabrication of Porous Lead Bromide Films by Introducing Indium Tribromide for Efficient Inorganic CsPbBr3 Perovskite Solar Cells
Source: Nanomaterials (Basel). 2021 May 11;11(5):1253. doi: 10.3390/nano11051253 (PMC8151783; doi:10.3390/nano11051253)
Supplement: Supplementary file 1 [file nanomaterials-11-01253-s001.zip › nanomaterials-1211784-supplementary.pdf]

Supplementary Materials

# Fabrication of Porous Lead Bromide Films by Introducing Indium Tribromide for Efficient Inorganic $CsPbBr_3$ Perovskite Solar Cells

**Xianwei Meng [1], Kailin Chi [2,*], Qian Li [3], Bingtao Feng [1], Haodi Wang [4], Tianjiao Gao [4], Pengyu Zhou [2], Haibin Yang [1] and Wuyou Fu [1,*]**

[1] State Key Laboratory of Superhard Materials, College of Physics, Jilin University, Changchun 130012, China; xwmeng17@mails.jlu.edu.cn (X.M.); fengbt20@mails.jlu.edu.cn (B.F.); yanghb@jlu.edu.cn (H.Y.)

[2] School of Science, Northeast Electric Power University, Jilin 132012, China; 20162715@neepu.edu.cn

[3] Beijing Key Lab of Cryo-Biomedical Engineering and Key Lab of Cryogenics, Technical Institute of Physics and Chemistry, Chinese Academy of Sciences, Beijing 100190, China; liqian@mail.ipc.ac.cn

[4] College of Physics, Jilin University, Changchun 130012, China; hdwang20@mails.jlu.edu.cn (H.W.); gaotj19@mails.jlu.edu.cn (T.G.)

* Corresponding: kailinchi@neepu.edu.cn (K.C.); fuwy@jlu.edu.cn (W.F.); Tel./Fax: +86-0432-64806674 (K.C.); +86-431-8516-8763-801 (W.F.)

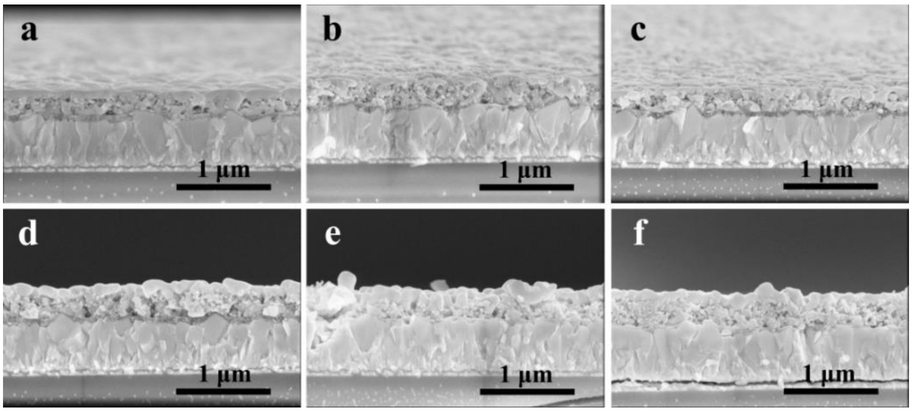


**Figure S1.** Cross-sectional SEM images of $PbBr_2$ films by introducing different concentrations of $InBr_3$: (**a**) 0.00 M; (**b**) 0.03 M; (**c**) 0.09 M; (**d**) 0.15 M; (**e**) 0.21 M; (**f**) 0.27 M.

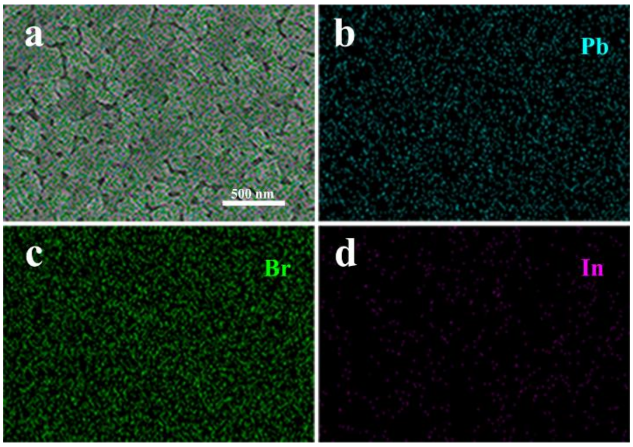


**Figure S2.** The SEM image of $InBr_3$:$PbBr_2$ film (**a**) and the corresponding EDS mapping of Pb (**b**), Br (**c**) and In (**d**).

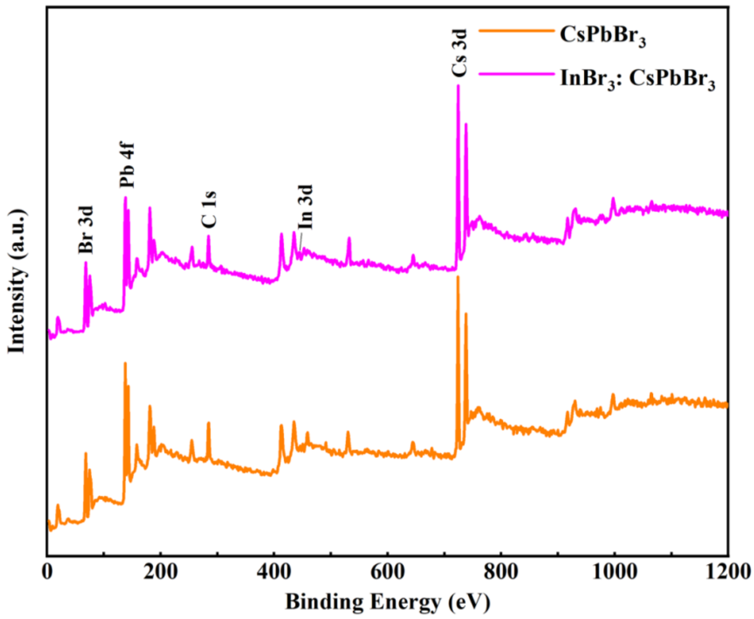


**Figure S3.** XPS spectra of $InBr_3$:$CsPbBr_3$ film.

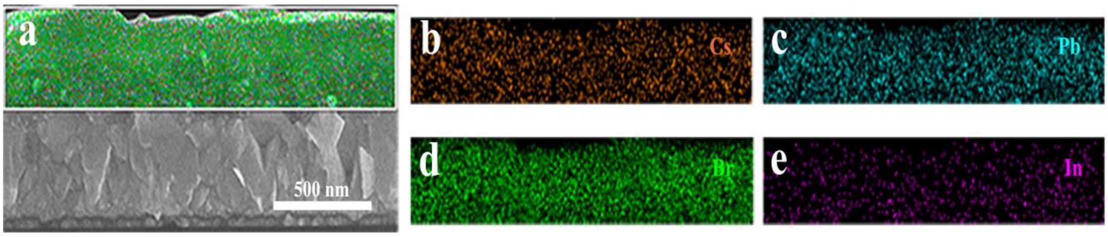


**Figure S4.** The cross-sectional SEM image of $InBr_3$:$CsPbBr_3$ film (**a**) and the corresponding EDS mapping of Cs (**b**), Pb (**c**), Br (**d**) and In (**e**).

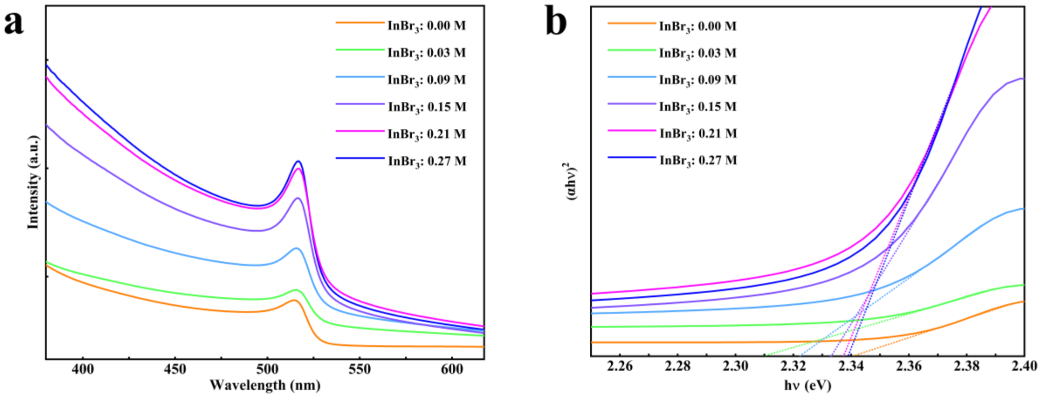


**Figure S5.** UV-vis absorption spectra (**a**) and $(\alpha h\nu)^2$ vs. hν plots (**b**) of the modules by introducing different concentrations of $InBr_3$.

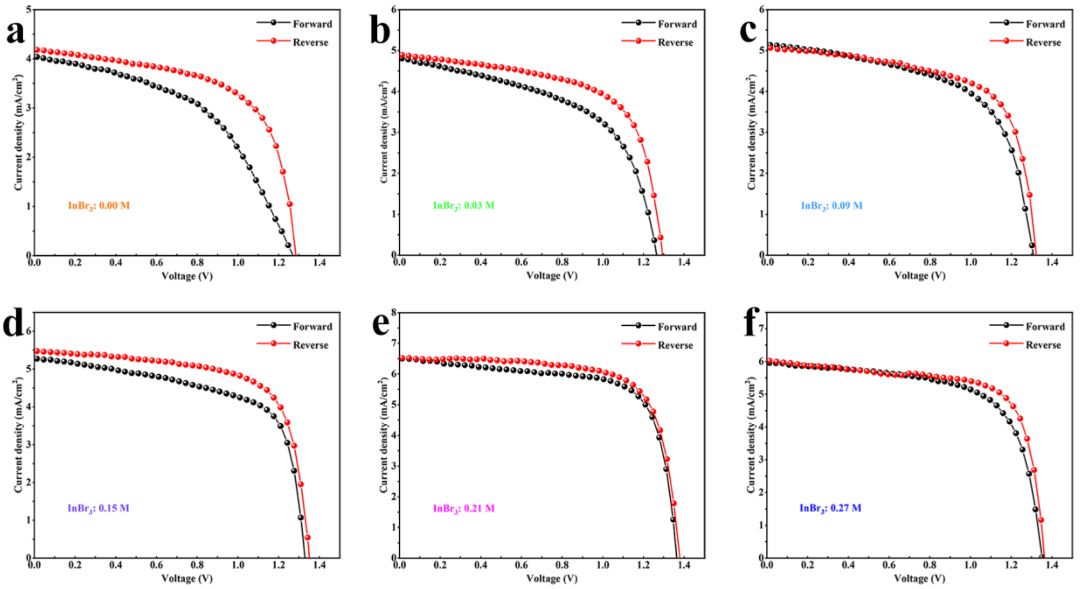


**Figure S6.** *J*–*V* curves with forward and reverse voltage scanning for the $InBr_3$:$CsPbBr_3$ devices: (**a**) 0.00 M; (**b**) 0.03 M; (**c**) 0.09 M; (**d**) 0.15 M; (**e**) 0.21 M; (**f**) 0.27 M.

**Table S1.** Electrochemical Impedance Spectroscopy parameters of the pristine and $InBr_3$ (0.21 M):$CsPbBr_3$ modules.

| Samples | $R_S$ (Ω) | $R_{rec}$ (Ω) | CPE-T (F) | CPE-P (F) |
|---|---|---|---|---|
| $CsPbBr_3$ | 22.72 | 765.20 | $8.95 \times 10^{-9}$ | 0.96 |
| $InBr_3$ (0.21 M):$CsPbBr_3$ | 20.26 | 1152.30 | $6.80 \times 10^{-9}$ | 0.98 |
